# The Membrane Fusion Step of Vaccinia Virus Entry Is Cooperatively Mediated by Multiple Viral Proteins and Host Cell Components

**DOI:** 10.1371/journal.ppat.1002446

**Published:** 2011-12-15

**Authors:** Jason P. Laliberte, Andrea S. Weisberg, Bernard Moss

**Affiliations:** Laboratory of Viral Diseases, National Institute of Allergy and Infectious Diseases, National Institutes of Health, Bethesda, Maryland, United States of America; University of Alberta, Canada

## Abstract

For many viruses, one or two proteins allow cell attachment and entry, which occurs through the plasma membrane or following endocytosis at low pH. In contrast, vaccinia virus (VACV) enters cells by both neutral and low pH routes; four proteins mediate cell attachment and twelve that are associated in a membrane complex and conserved in all poxviruses are dedicated to entry. The aim of the present study was to determine the roles of cellular and viral proteins in initial stages of entry, specifically fusion of the membranes of the mature virion and cell. For analysis of the role of cellular components, we used well characterized inhibitors and measured binding of a recombinant VACV virion containing Gaussia luciferase fused to a core protein; viral and cellular membrane lipid mixing with a self-quenching fluorescent probe in the virion membrane; and core entry with a recombinant VACV expressing firefly luciferase and electron microscopy. We determined that inhibitors of tyrosine protein kinases, dynamin GTPase and actin dynamics had little effect on binding of virions to cells but impaired membrane fusion, whereas partial cholesterol depletion and inhibitors of endosomal acidification and membrane blebbing had a severe effect at the later stage of core entry. To determine the role of viral proteins, virions lacking individual membrane components were purified from cells infected with members of a panel of ten conditional-lethal inducible mutants. Each of the entry protein-deficient virions had severely reduced infectivity and except for A28, L1 and L5 greatly impaired membrane fusion. In addition, a potent neutralizing L1 monoclonal antibody blocked entry at a post-membrane lipid-mixing step. Taken together, these results suggested a 2-step entry model and implicated an unprecedented number of viral proteins and cellular components involved in signaling and actin rearrangement for initiation of virus-cell membrane fusion during poxvirus entry.

## Author Summary

Poxviruses are large DNA viruses that cause diseases in humans and other animals. To initiate infection, the core of the large, membrane-enveloped particle must penetrate into the cytoplasm where replication occurs. For most enveloped viruses only one or two proteins are needed for attachment and penetration. However, at least sixteen poxvirus proteins are dedicated to entry: four for attachment and twelve for penetration. The latter proteins form the entry fusion complex (EFC) and are conserved in all poxviruses indicating that the entry mechanism has been retained since the origin of the family. The purpose of the present study was to determine the cellular processes and poxviral proteins needed for fusion of the viral and cellular membranes. We found that a variety of inhibitors that interfered with cell signaling and reorganization of the actin cytoskeleton prevented membrane fusion as determined by lipid mixing, whereas others targeted the subsequent stage in entry. In addition, seven viral protein components of the EFC were required for the initial membrane fusion step, whereas three were not. A neutralizing monoclonal antibody to one of the latter also did not interfere with membrane lipid mixing but still prevented core entry supporting a 2-step poxvirus entry model.

## Introduction

Entry of enveloped viruses into cells can be divided into three steps: (i) close apposition of viral and cellular membranes, (ii) lipid mixing of the outer membrane leaflets leading to formation of a hemifusion intermediate, and (iii) formation and expansion of a fusion pore allowing entry of the viral nucleoprotein or core into the cytoplasm [1]. One or two glycoproteins that provide cell binding and membrane fusion are sufficient to mediate entry of many enveloped viruses [2]. The process is more complex for members of the herpesvirus family, which employ four to five glycoproteins for entry [3]. Poxviruses represent an extreme case, as at least sixteen unglycosylated vaccinia virus (VACV) proteins participate in this process (referenced below). The large number of poxvirus proteins and the absence of any that resemble conventional membrane fusion proteins by sequence suggest a novel entry mechanism. For mature virions (MVs), the basic and most abundant infectious VACV particle, entry can occur by fusion at the plasma membrane [4], [5] or in a low pH-dependent manner from within an intracellular vesicle, depending to some extent on the virus strain [6], [7] and cell type [7]–[9]. Endocytosis of MVs is believed to occur by macropinocytosis [10]–[15] or dynamin-mediated fluid phase uptake [16], consistent with a role for actin dynamics and cell signaling. Progeny virions that depart the cell by exocytosis contain an additional membrane that helps escape antibody neutralization and is ultimately ruptured to allow fusion of the enclosed MV with the plasma membrane or endocytic vesicle [17], [18].

Four VACV proteins are involved in attachment of MVs [19]–[22] and twelve, conserved in all members of the poxvirus family, participate in subsequent entry steps [23]–[34]. Initial binding to target cells occurs via interactions of the MV attachment proteins with cell surface glycosaminoglycans or laminin. A cellular protein, referred to as VACV penetration factor, appears to be important for entry but exactly how is not yet understood [16]. The twelve conserved VACV entry proteins are mostly small, ranging in size from 35 to 377 amino acids, and have a N- or C-terminal transmembrane domain. The proteins are all components of the MV membrane, which is formed within the cytoplasm by incompletely defined mechanisms rather than by budding as typically occurs with other viruses [35]. This feature, as well as the association of most or all the proteins in a complex [31], makes it difficult to investigate the roles of individual entry proteins. A useful approach has been to construct conditional lethal mutants, with one putative entry gene controlled by the *Escherichia coli lac* operator/repressor system and positively regulated by ß-D-isopropylthiogalactopyanoside (IPTG) inducer, or with an analogous tetracycline-inducible system. These mutants share similar phenotypes: in the presence of inducer, replication proceeds normally and the progeny virions contain the protein product of the inducible gene and are infectious; in the absence of inducer, progeny virions appear indistinguishable from wild type by electron microscopy and protein analysis (except for the missing entry protein) but have very low infectivity. Although the non-infectious virions bind to cells, immunofluorescence microscopy studies show reduced numbers of cores in the cytoplasm. With the exception of I2 [30], repressed expression of the individual proteins does not significantly reduce the trafficking of the others to the MV membrane. However, when expression of an individual component is repressed, the formation or stability of the complex is reduced, as determined by detergent extraction and immunoaffinity purification [31]. The proteins A16, A21, A28, G3, G9, H2, J5, L5 and O3, make up the central components of the so-called entry fusion complex (EFC). The L1 and F9 proteins are also required for entry; although they physically interact with the EFC, they are not required for assembly or stability of the complex, and consequently have been referred to as EFC-associated proteins [26], [32]. The overall structure of the EFC has not been elucidated, though several pair-wise protein interactions have been identified [36]–[38].

**Figure 1:**
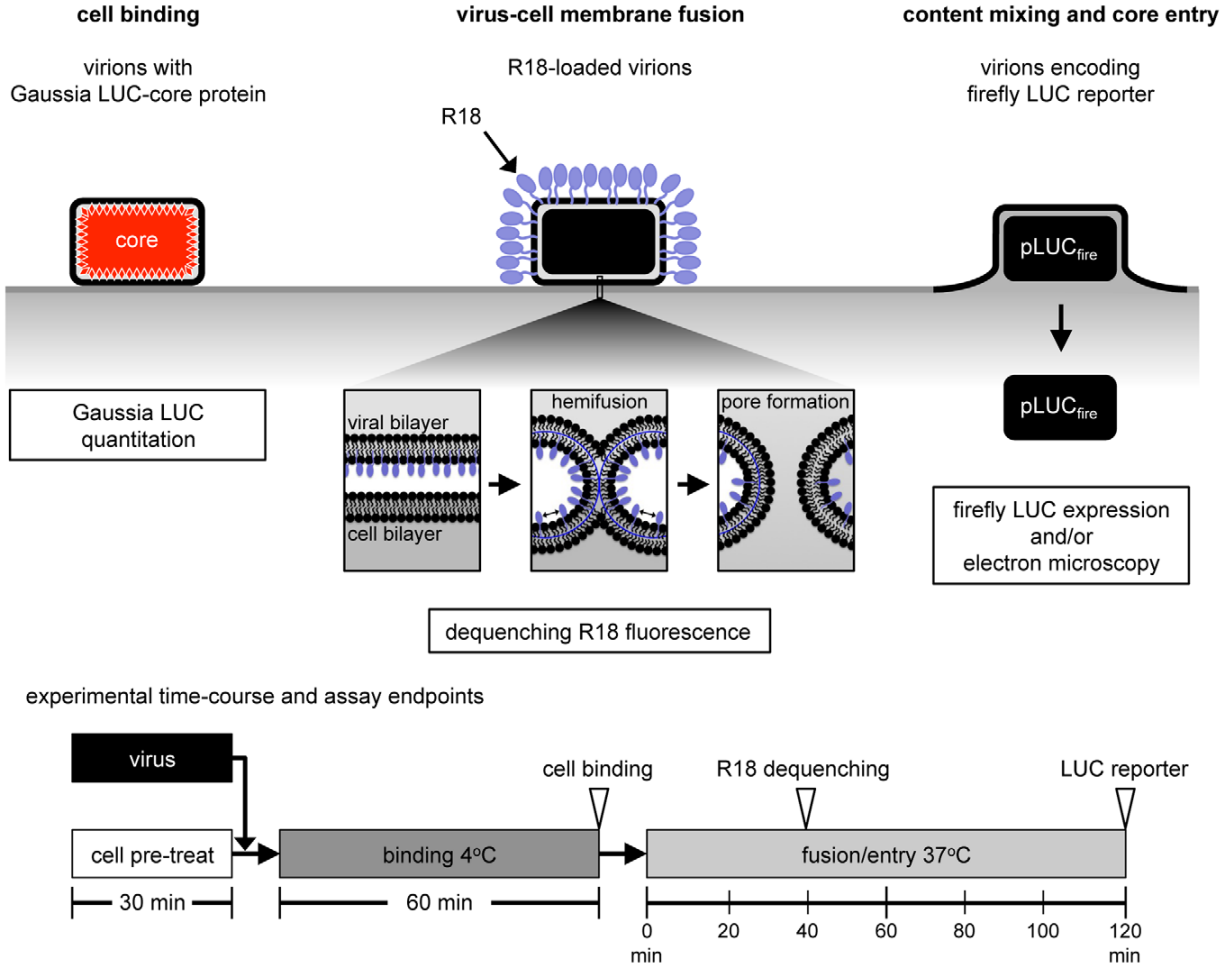
Virion binding, lipid mixing and core entry assays. VACV Gauss-A4, a recombinant VACV with Gaussia LUC fused to a core protein, was used to measure the binding of virions at 4°C by assaying cell-associated LUC activity. For virus-cell membrane fusion, R18-loaded virions were bound to target cells at 4°C, shifted to 37°C, and the dequenching of R18 due to lipid mixing was measured by increased fluorescence. WRvFire, a recombinant VACV that expresses firefly LUC under an early promoter, was used to infect cells and newly synthesized LUC was measured. Direct visualization of virions fusing with the plasma membrane and quantification of viral cores in the cytosol were achieved by transmission electron microscopy. The times used for pretreatment, binding and entry are depicted at the bottom of the figure.

The mechanisms involved in poxvirus entry are poorly understood. Previous studies have depended on post-membrane fusion assays and a specific role of the EFC in fusion could only be inferred from the inability of cells infected with the mutant viruses made in the absence of IPTG to undergo low pH-induced syncytia formation. Thus, direct evidence for a role of EFC proteins in membrane fusion during entry of virions has been lacking. Here, we used a variety of approaches including cell binding, membrane lipid mixing, core entry and reporter gene expression (Figure 1) to evaluate the roles of host components and individual MV membrane proteins.

## Results

### VACV-Cell Membrane Fusion and Core Entry

Fusion of viral and cellular membranes involves lipid mixing, which can be studied by loading a self-quenching fluorescent probe such as octadecylrhodamine (R18) into viral membranes (Figure 1). Fusion of viral and cell membranes results in dilution of the probe and increased fluorescence [39]. Dequenching does not require full fusion of the viral and cell membrane but can occur at the initial step in which only the outer leaflets of the viral and cellular membranes fuse, known as hemifusion [1]. Therefore, dequenching could signify the occurrence of hemifusion alone or full fusion with pore formation. In a 2-step membrane fusion model (see Discussion), inhibitors that prevent dequenching must operate at or prior to the hemifusion step, which precedes full fusion.

**Figure 2:**
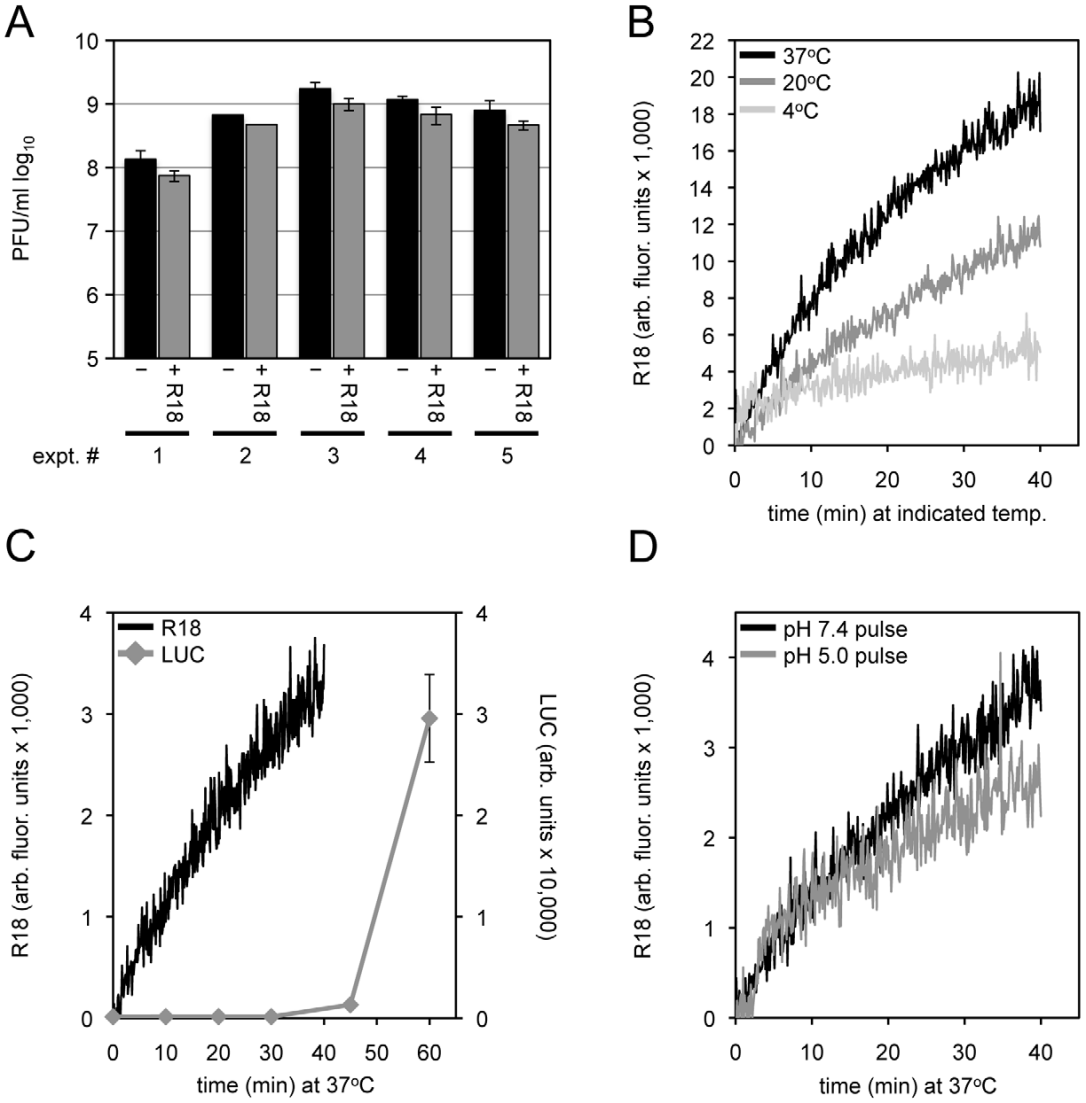
Membrane fusion and core entry. (A) Equivalent numbers of purified MVs were untreated or loaded with R18 for 20 min at room temperature. Unbound R18 was removed by pelleting and washing the virus. Control and R18-labeled virions were resuspended and serial dilutions made to assay virus infectivity (PFU/ml) by plaque assay. The results of five independent experiments with error bars are plotted. (B) Purified R18-loaded MV particles were bound to HeLa cells at 4°C for 60 min. The cells were then incubated at 4°C, 20°C, or 37°C for 40 min while R18 fluorescence was monitored and quantified as arbitrary fluorescent units. (C) R18-loaded MVs (recombinant WRvFire) were incubated with HeLa cells at 4°C to permit binding. Washed cells were then placed in a cuvette containing pre-warmed media at 37°C and fluorescence was monitored over time (black line; left *y*-axis). In parallel, unlabeled MVs were bound to cells in the cold and then shifted to 37°C. Cell lysates were prepared at indicated times and assayed for LUC activity (gray line; right *y*-axis). (D) An equivalent number of purified R18-loaded MVs were bound to HeLa cells in the cold for 60 min. Virus-bound cells were then placed at 37°C in a pre-warmed cuvette containing media adjusted to either pH 7.4 or 5.0 while R18 fluorescence was monitored. After 3 min, cell media was adjusted back to neutral \ and R18 fluorescence monitoring continued.

In the present experiments, sucrose gradient purified VACV MVs were incubated with R18 at room temperature for 20 min. Incorporation of R18 into MVs minimally affected infectivity as shown in Figure 2A. After removal of excess R18, the MVs were incubated with HeLa cells for 1 h at 4°C to allow adsorption and then the temperature was raised to permit fusion. R18 fluorescence was more rapid at the physiological temperature of 37°C than at 20°C (Figure 2B), consistent with an active transfer process. We used WRvFire, a recombinant VACV that expresses firefly luciferase (LUC) regulated by an early promoter, to compare the kinetics of fusion and reporter gene expression. Whereas fusion occurred within a few minutes after incubation of virus-bound cells, LUC expression was detected at 40 min (Figure 2C) and was routinely assayed after 1 or 2 h.

The above results supported the use of the fluorescent R18 probe for analyzing VACV-cell membrane fusion. In subsequent experiments we compared the effects of inhibitors on binding of virions to cells, fusion, and core entry as measured by LUC expression and in some cases by transmission electron microscopy.

### Fusion Was Not Enhanced by Low pH or Greatly Reduced by Cholesterol Depletion

**Figure 3:**
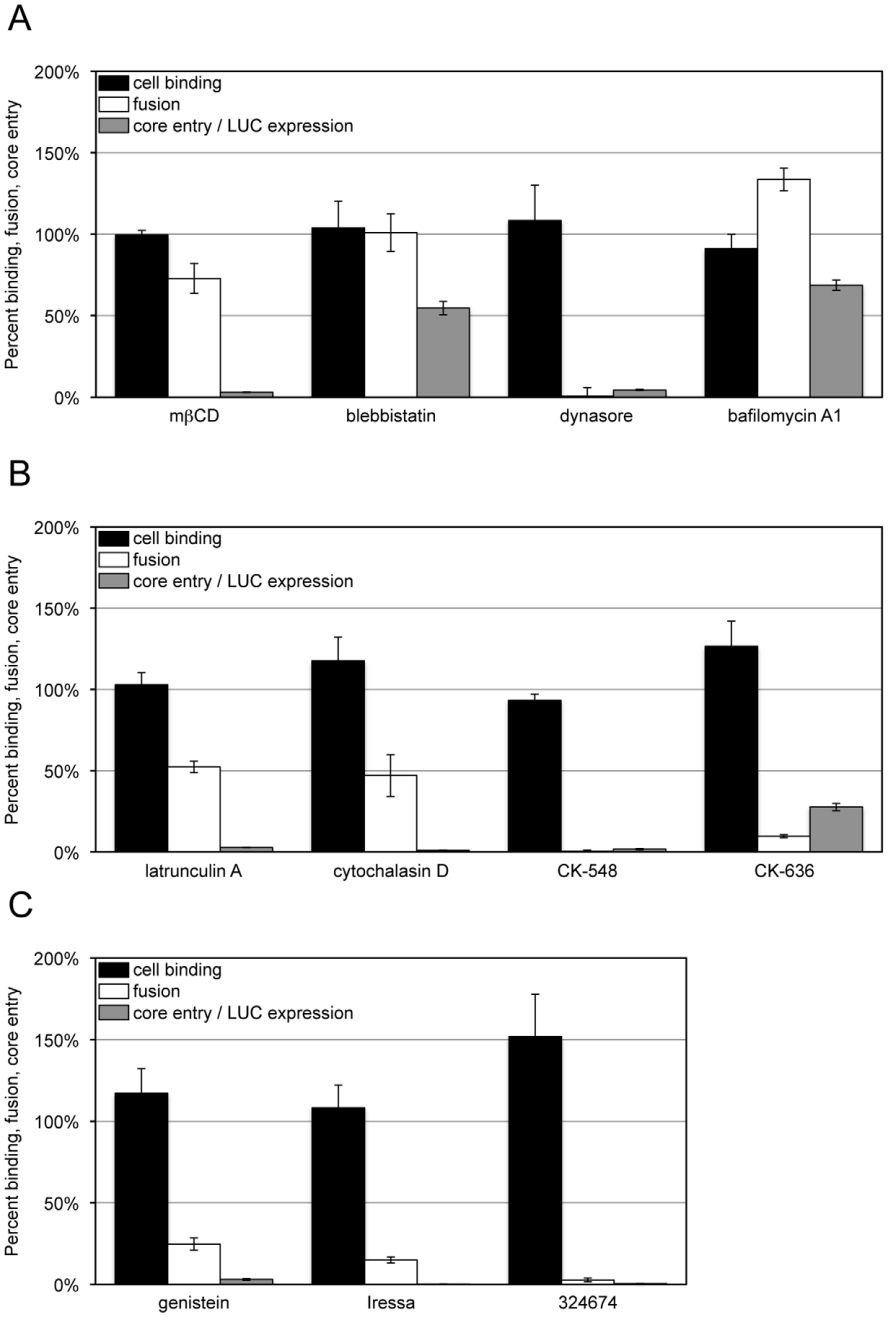
Effects of inhibitors on VACV-cell attachment, membrane fusion and core entry. HeLa cells were left untreated or pre-treated for 30 min at 37°C with: (A) mßCD (10 mM), blebbistatin (75 µM), dynasore (100 µM) and bafilomycin A1 (50 nM); (B) latrunculin A (10 µM), cytochalasin D (10 µM), CK-548 (100 µM) and CK-636 (100 µM); (C) genestein (100 µM), Iressa (40 µM), and 32674 (40 µM). For cell binding (black bars), control and inhibitor-treated cells were incubated with equivalent numbers of VACV Gauss-A4 MVs at 4°C for 60 min. Unbound virions were removed by washing and cells lysed to measure cell-associated Gaussia LUC activity. For membrane fusion (white bars), control and inhibitor-treated cells were incubated with equivalent numbers of R18-loaded WRvFire particles at 4°C for 60 min. Washed cells were then incubated at 37°C for 40 min in the presence of the indicated inhibitor while R18 fluorescence was monitored. For core entry (gray bars), equivalent numbers of WRvFire MVs were adsorbed to control and inhibitor-treated cells at 4°C for 60 min. Cells were washed and incubated for 2 h at 37°C in the presence or absence of the indicated inhibitor. Cells were then lysed and firefly LUC activity in cell extracts measured. Data are represented as percent of the untreated cell control for each assay.

An earlier study had shown that fusion of VACV strain WR was not enhanced at low pH [40], which in retrospect seemed surprising in view of the subsequent demonstration of low pH enhancement of core entry and reporter gene expression [6]. Nevertheless, we confirmed the similar rates of VACV WR fusion following a brief incubation with a pH 7.4 or pH 5.0 buffer and return to neutral pH (Figure 2D). Furthermore, we found that bafilomycin A1, which prevents endosomal acidification and reduces firefly LUC expression, had little effect on binding of MVs containing a Gaussia LUC core protein chimera or membrane fusion (Figure 3A), similar to previous findings of membrane fusion in the presence of ammonium chloride and chloroquine [40]. Thus, low pH promotes an entry step beyond membrane lipid mixing.

Depletion of cellular cholesterol reversibly prevents the accumulation of VACV cores in the cytosol at a post-attachment step [41]. Treatment of HeLa cells with methyl- ß-cyclodextrin (mßCD) resulted in up to a 74% reduction in total cellular cholesterol levels (Figure S1A) without reducing cell viability over the time-course of the experiment (Figure S1B), although some cell rounding occurred. Nevertheless, MVs efficiently bound to cholesterol-depleted HeLa cells and R18 fluorescence was only mildly reduced, whereas LUC expression was greatly inhibited (Figure 3A). These data indicated that the lowered level of cellular cholesterol was sufficient for membrane lipid mixing but impaired a later step in entry or reporter gene expression.

### Cellular Components Required for Virion Attachment, Membrane Fusion and Core Entry

Inhibitors targeting membrane blebbing, dynamin function, actin dynamics, and the activities of certain protein kinases have been shown to reduce VACV entry to varying extents as measured by reporter gene expression or detection of cytoplasmic cores [11]–[13], [16], [42]. In the present experiments, HeLa cells were preincubated for 30 min with inhibitors at previously used concentration ranges and the drugs were maintained in the medium during and after virus adsorption. Infection with VACV induces actin-enriched protrusions or cellular blebs [42] and entry can be partially reduced by blebbistatin, a small molecule specific inhibitor of myosin-II-dependent blebbing, virus movement along filopodia and macropinocytosis [11], [43], [44]. Blebbistatin was without effect on virion attachment but reduced LUC reporter expression by about 50% (Figure 3A), similar to the value previously reported for a GFP reporter assay [11]. However, we found little or no effect on dequenching of the R18 probe (Figure 3A), indicating that membrane fusion can occur independently of cell membrane blebbing.

Dynasore is a small molecule inhibitor of the GTPase activity of dynamin1, dynamin2 and the mitochondrial dynamin and is a rapid and potent inhibitor of dynamin-dependent endocytic pathways [45]. Dynamin also directly interacts with actin and regulates the actin cytoskeleton [46]–[48]. The effect of dynasore on VACV entry is ambiguous as it was reported not to influence entry in some studies [11] but to inhibit entry in another [16]. We found that dynasore had no effect on virion binding to HeLa cells but severely decreased LUC expression (Figure 3A). Moreover, dynasore potently inhibited membrane fusion (Figure 3B). These results implicated cellular dynamin as a critical factor in promoting VACV entry into HeLa cells at the membrane fusion step.

We also tested several specific inhibitors of actin dynamics: CK-636 and CK-548 bind to the Arp2/3 complex and prevent actin nucleation whereas latrunculins and cytochalasins bind actin and inhibit polymerization [49], [50]. These drugs had little effect on virion attachment but severely blocked LUC expression (Figure 3B). CK-548 and CK-636 were also very effective inhibitors of membrane fusion, whereas latrunculin A and cytochalasin D inhibited fusion by approximately 50% at the concentrations used (Figure 3B). These studies indicated a role for actin rearrangement in membrane fusion and raised the possibility that the effect of dynasore was related to its influence on the actin cytoskeleton rather than endocytosis.

Cell signaling has been reported to have a role in VACV entry at the stage of blebbing and macropinocytosis [11]. Genestein, gefitinib (Iressa) and 324674 (PD153035) are small molecule tyrosine kinase inhibitors [51], [52]. These drugs did not reduce virion binding but profoundly inhibited LUC expression (Figure 3C). Moreover, they also greatly inhibited membrane fusion (Figure 3C). The results could be related to the relative specificity of gefitinib and 324674 for epidermal growth factor receptor signaling, which causes rapid actin polymerization and rearrangement [53].

Based on a previous report [11], we attempted to bypass the effects of inhibitors of actin remodeling and signaling on entry by brief low pH treatment of cells with attached virions. However, in our hands, such treatments only alleviated the effects of drugs such as bafilomycin A1, concanamycin and monensin that prevented endosomal acidification [6] but did not bypass the effects of several other inhibitors on entry as measured by LUC expression or R18 dequenching (Figure S2).

**Figure 4:**
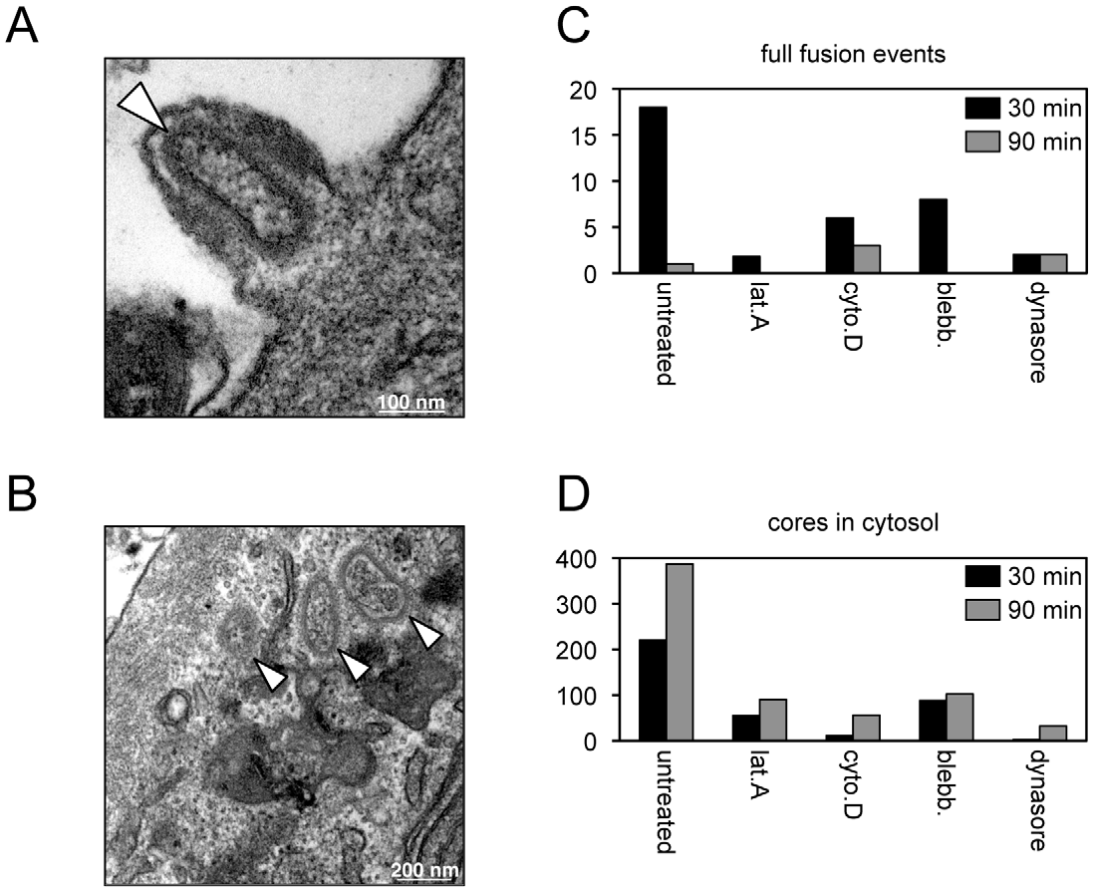
Effects of inhibitors on entry determined by transmission electron microscopy. Purified MVs (350 PFU per cell) were spinoculated onto inhibitor-treated HeLa monolayers at 4°C for 60 min. Virus-bound cells were then incubated for either 30 or 90 min at 37°C in the presence or absence of the indicated inhibitor, fixed and processed for transmission electron microscopy. Representative images from untreated cells at 30 min showing full fusion of virion and plasma membranes resulting in pore formation (A) and cores in the cytosol (B). White arrowheads point to cores; scale bars indicate magnification. For each infection, a total of 90 randomly-selected cell sections were visualized and the number of plasma membrane full fusion events (C) and viral cores in the cytosol (D) were determined at 30 and 90 min.

Core entry steps were also analyzed by transmission electron microscopy. The results cannot be precisely compared to the above assays because a high virus multiplicity and spinoculation were used to allow counting of a sufficient number of virus particles in thin sections of infected cells. Hemifusion cannot be detected by this procedure and the earliest recognizable entry step consisted of full fusion of the viral and plasma membranes with an open pore allowing core entry (Figure 4A). Although MVs can be readily detected in vesicles, full fusion of viral and vesicle membranes are rarely seen (5). Cores that accumulate in the cytoplasm (Figure 4B) could have entered through the plasma membrane or an endocytic vesicle. In the absence of inhibitors, the number of plasma membrane full fusion images decreased and cores in the cytoplasm increased between 30 and 90 min (Figure 4C, D). At both times, the numbers of plasma membrane full fusion images (Figure 4C) and cytoplasmic cores (Figure 4D) were reduced when the cells were treated with blebbistatin, dynasore, latrunculin A or cytochalasin D. These observations confirmed the results obtained with the LUC assay for measuring core entry.

In summary, our data are generally consistent with other studies showing the importance of cell signaling and remodeling of the actin cytoskeleton on VACV entry [10]–[15], and importantly further demonstrate that these activities are necessary for the membrane fusion step. Low pH, cholesterol and membrane blebbing appear to be more important for entry steps beyond membrane lipid mixing.

### Roles of EFC Proteins in Virus-Cell Membrane Fusion

**Figure 5:**
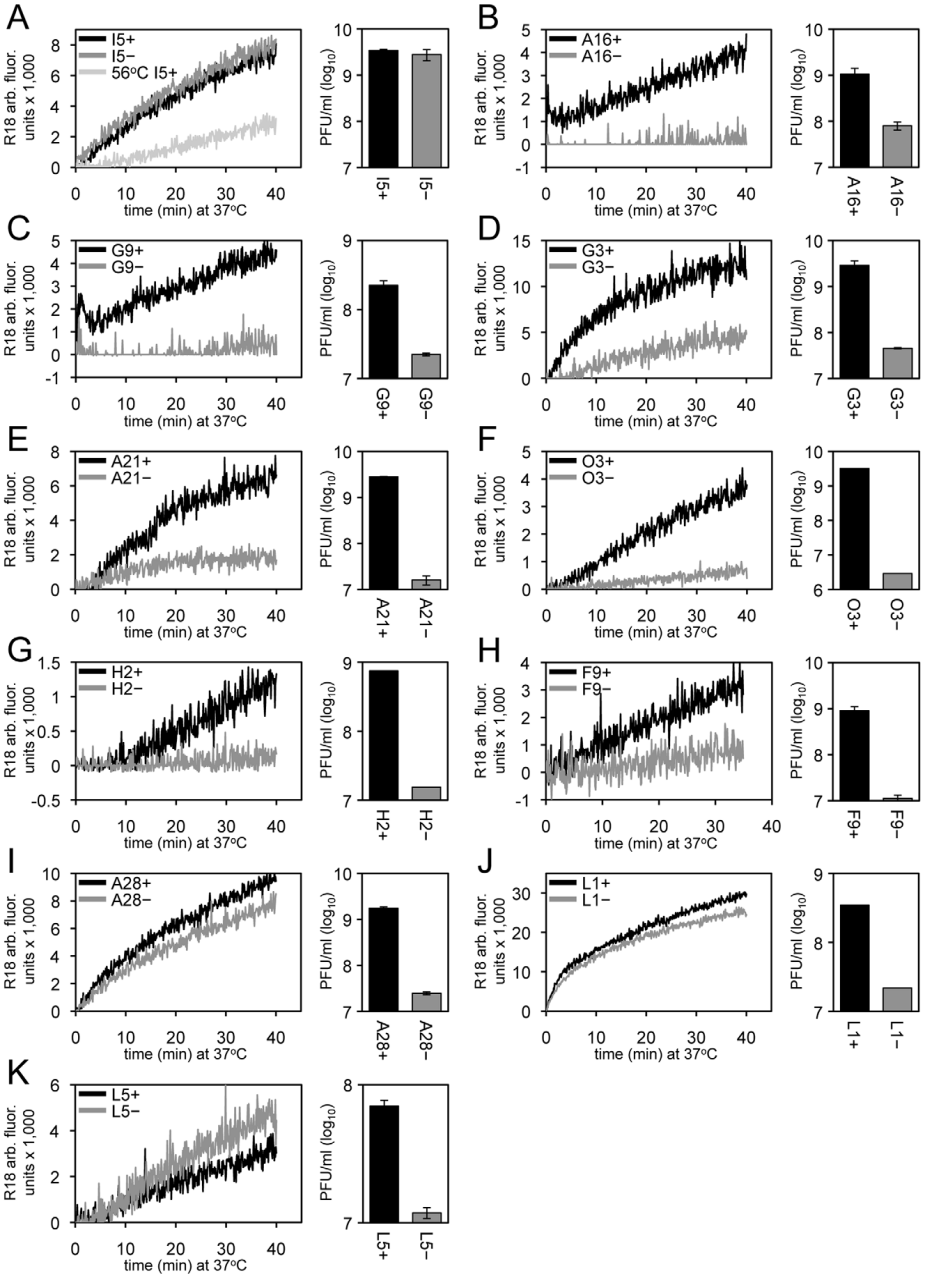
Effects of deficiencies of individual virion membrane proteins on membrane fusion and virus infectivity. In each panel, equivalent numbers of purified R18-loaded MVs were bound to HeLa cells at 4°C for 60 min and unbound virions were removed by washing. Virus-bound cells were then incubated at 37°C for 40 min and R18 fluorescence was monitored and plotted as arbitrary units. Parallel cultures were incubated for 48 h and the yield of virus was determined by plaque assay. Recombinant viruses were as follows: (A) ΔI5L, (B) IPTG-inducible A16, (C) IPTG-inducible G9, (D) IPTG-inducible G3, (E) IPTG-inducible A21, (F) IPTG-inducible O3, (G) IPTG-inducible H2, (H) IPTG-inducible F9, (I) IPTG-inducible A28, (J) IPTG-inducible L1, and (K) IPTG-inducible L5. For panels B-K, the plus and minus signs in the upper left signifies the virus was grown in the presence or absence of IPTG, respectively. For panel A, the plus and minus refer to wild type virus and a deletion mutant, respectively. As a negative control, 56°C heat-inactivated I5+ virions (panel A) were assayed for hemifusion and infectivity (<10^5^ PFU/ml; data not shown).

Most or all of the MV membrane proteins required for entry, as distinguished from cell attachment, are components of the EFC (A16, A21, A28, G3, G9, H2, J5, L5, O3) or physically associated with the EFC (L1, F9). We employed conditional lethal mutants for all EFC and EFC-associated proteins except J5, for which a stringent mutant was unavailable. As a control, we tested a mutant with a deletion of the gene encoding the I5 MV membrane protein that is not required for entry [54]. The recombinant viruses were replicated in the presence or absence of the IPTG inducer and the MVs were purified by sucrose gradient sedimentation. For each mutant, the number of purified virions was determined from the optical density. In some cases, virions were inactivated at 56°C prior to adsorption to cells as an additional control [55]. Equivalent numbers of particles were loaded with R18 and washed by sedimentation to remove excess dye. Dye transfer to HeLa cells was determined by increased fluorescence as in the preceding sections. In addition parallel cultures were maintained for 48 h and the yield of infectious virus determined by plaque assay. As expected, R18-loaded MVs lacking the I5 protein (I5^−^) promoted R18 probe transfer as efficiently as wild type MV (I5^+^), whereas transfer was reduced with the heat-inactivated MVs (Figure 5A). Virions deficient in individual EFC and EFC-associated proteins had very low infectivity and except for A28, L1 and L5 mutants exhibited severely reduced R18 dequenching as well (Figure 5B-K), providing the first evidence of a direct role of EFC proteins in the membrane fusion step of virus entry. Previous studies had only shown that the EFC was required for fusion of infected cells.

**Figure 6:**
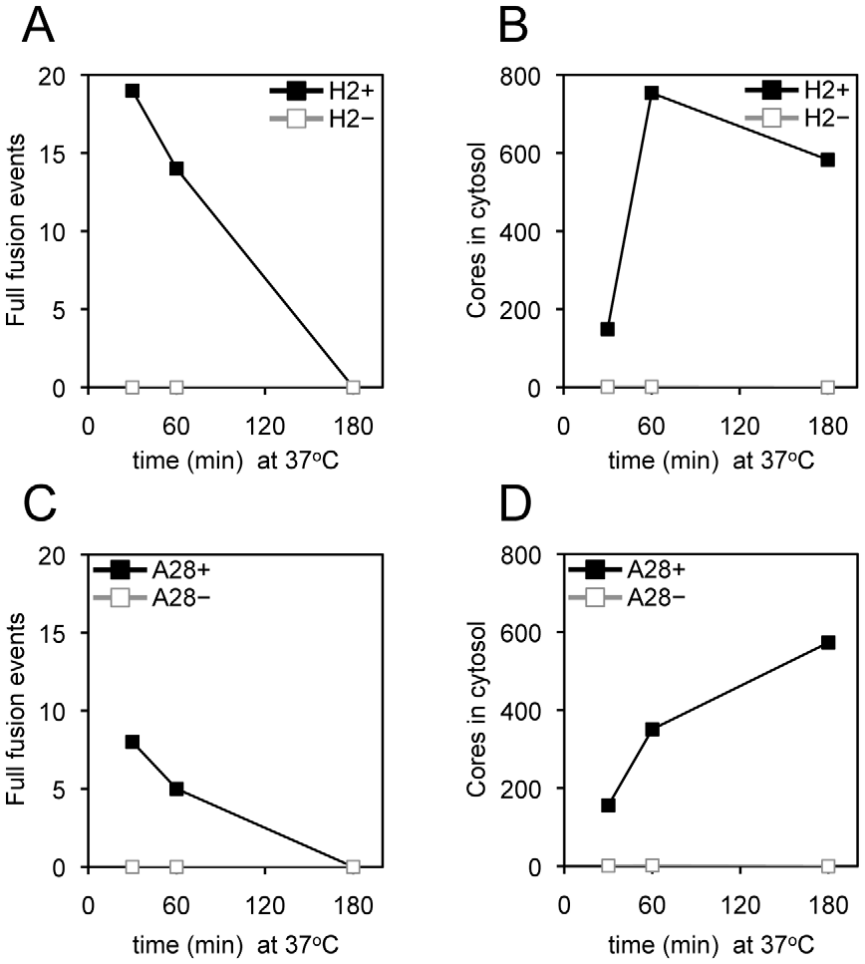
Entry of virions lacking H2 or A28 protein determined by transmission electron microscopy. Purified MVs (350 PFU per cell or equivalent number of particles) possessing (+) or lacking (-) H2 or A28 protein as indicated were spinoculated onto pre-chilled HeLa cell monolayers at 4°C for 60 min. Virus-bound cells were then incubated for either 30, 60 or 180 min at 37°C, fixed and processed for electron microscopy. For each infection, a total of 90 randomly-selected cell sections were inspected and the number of full fusion events (A and C) and free viral cores in the cytosol (B and D) were determined as described in the legend to Figure 4.

We used transmission electron microscopy to monitor core entry steps, following attachment of H2^+^, H2^−^, A28^+^ and A28^−^ virions. We chose H2 and A28 as examples of mutants that reduced and allowed R18 dequenching, respectively (Figure 5G, I). As indicated earlier, a high multiplicity and spinoculation was needed because of the thin cell sections. The lower numbers of full fusions with pore formation at the plasma membrane and cytoplasmic cores in cells infected with H2^−^ virions compared to H2^+^ virions were expected in view of the inability of the former to mediate R18 dequenching (Figure 6A,B). However, there was a similar reduction in full fusion images at the plasma membrane and cytoplasmic cores after infection with A28^−^ virions compared to A28^+^ virions (Figure 6C, D) despite the greater ability of the former to allow membrane fusion as determined by lipid mixing. Inhibition of core entry was previously shown using a confocal microscopy assay for virions deficient in L1 [32] and L5 [34] confirming an entry block despite their ability to allow lipid mixing as shown here.

### Neutralizing Antibody to L1 Inhibits Entry at a Step Beyond Membrane Lipid Mixing

**Figure 7:**
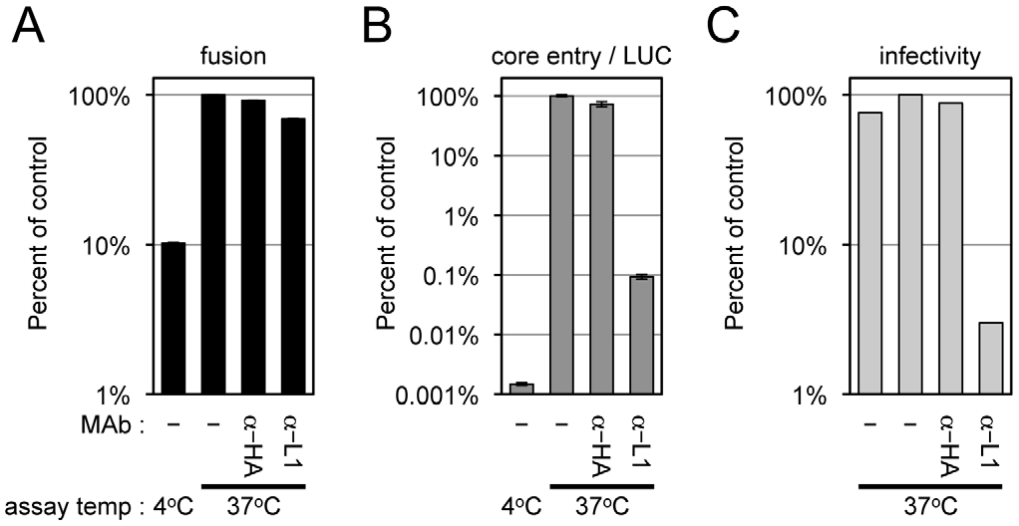
Effects of anti-L1 MAb on virus-cell membrane fusion, viral core entry and virus infectivity. Equivalent numbers of R18-loaded virions (WRvFire) were incubated with or without 100 µg/ml of anti-L1 mouse MAb or control anti-HA mouse MAb for 30 min at room temperature. Virions were then assayed for their ability to mediate virus-cell membrane fusion by R18 dequenching (A) or core entry by LUC expression (B), at either 37°C or 4°C. Infectivity (C) was assayed by adsorbing each virus sample at 37°C to BS-C-1 monolayers for 60 min and enumerating plaque formation 48 h later. Data are represented as percent of the no MAb control at 37°C for each assay.

The above results showing that L1-deficient virions allowed membrane fusion but not core entry led us to investigate the effect of a potent L1-neutralizing monoclonal antibody (MAb) [56]. We found that a concentration of L1 MAb that severely inhibited core entry as determined by LUC expression and formation of infectious virus had minimal effect on membrane fusion as determined by R18 dequenching (Figure 7). This result was confirmed by a flow cytometry-based 1,1′-dioctadecyl-3,3,3′,3′-tetramethylindodicarbocyanine (DiD) lipid mixing assay using a wide-range of MAb concentrations (Figure S3).

### Attempt to Trans-Complement an EFC Mutant

**Figure 8:**
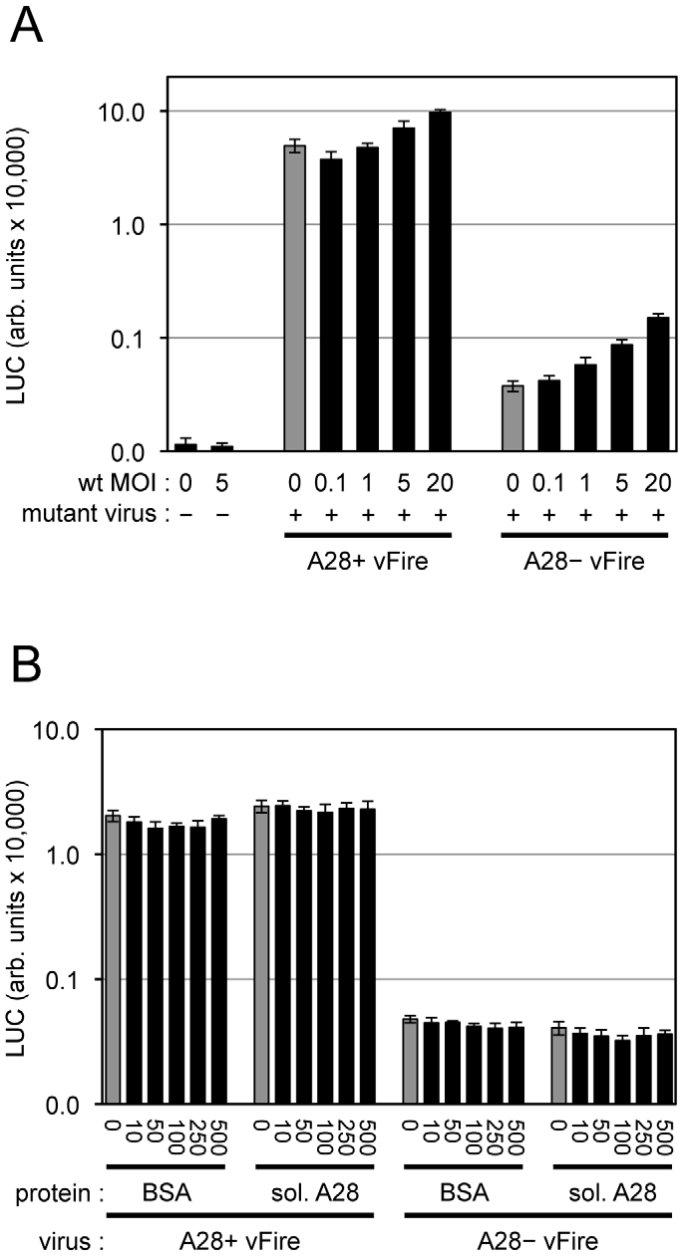
Attempt to trans-complement entry of virions lacking A28. (A) Equivalent numbers of purified A28^+^ or A28^−^ MVs expressing firefly LUC (WRvFire) were mixed with varying amounts of purified, wild type (wt) MVs as indicated. Virions were adsorbed to HeLa cell monolayers at 4°C for 60 min. Cells were washed and placed at 37°C for 2 h to allow virus entry. Cells were then lysed and firefly LUC activity measured. MOI (multiplicity of infection; PFUs per cell) of wt VACV is indicated. (B) Equivalent numbers of purified A28^+^ or A28^−^ MVs expressing firefly LUC were mixed with varying amounts of bovine serum albumin (BSA) or soluble A28 protein as indicated (ng/ml). Virions were adsorbed to HeLa cell monolayers at 4°C for 60 min. Cells were washed and placed at 37°C for 2 h to allow for virus entry. Cells were then lysed and LUC activity measured.

We still needed to consider the possibility that the role of the EFC is to activate the cell for virion entry rather than to directly participate in the entry step per se. In this context, Mercer and Helenius [11] had reported that very few VACV particles are needed to induce widespread blebbing and actin rearrangement. To further investigate the role of the EFC in entry, we coinfected cells with wild type VACV and either A28^+^ or A28^−^ virions that expressed firefly LUC. We used a particle/cell multiplicity of approximately 200 for the A28^+^ and A28^−^ virions and varied the multiplicity of the wild type virions from 9 to 1840 particles/cell (equivalent to 0.1 to 20 plaque forming units (PFU)/cell). Coinfection with wild type virions caused a two-fold increase in LUC expression by A28^+^ virions and raised expression about four-fold for A28^−^ virions (Figure 8A). However, the latter was still only 3% of the value for A28^+^ virions indicating that efficient trans-complementation had not occurred. We also determined that soluble A28 protein [57] mixed with virions had no effect on entry of either the A28^+^ or A28^−^ virions (Figure 8B).

## Discussion

Viral and cellular membranes each consists of two leaflets and in principal membrane fusion could occur by two different pathways as discussed by Chernomordik [1]. The direct fusion model posits that pores form in each of the apposing membranes and the pore rims join forming a fusion pore that allows lipid and content mixing in a single step. In contrast, the 2-step model posits fusion of the outer leaflets of the apposing membranes to form a hemifusion intermediate followed by merging of the inner leaflets to form the fusion pore. In the latter model, lipid mixing and content mixing occur sequentially. Evidence to support the second model involving a hemifusion intermediate has been obtained for several different viruses by demonstrating membrane lipid mixing without content mixing by mutation of viral fusion proteins, slowing or interrupting fusion with inhibitors and decreasing the surface density of viral fusion proteins [58]–[61]. In the present study of VACV, we showed that membrane lipid mixing could occur without core entry under three circumstances: depletion of certain EFC proteins (A28, L1 or L5), neutralization of VACV with a MAb to the L1 EFC-associated protein, and partial cholesterol depletion of the cell membrane. These findings are consistent with a 2-step entry model with a hemifusion intermediate for VACV.

**Figure 9:**
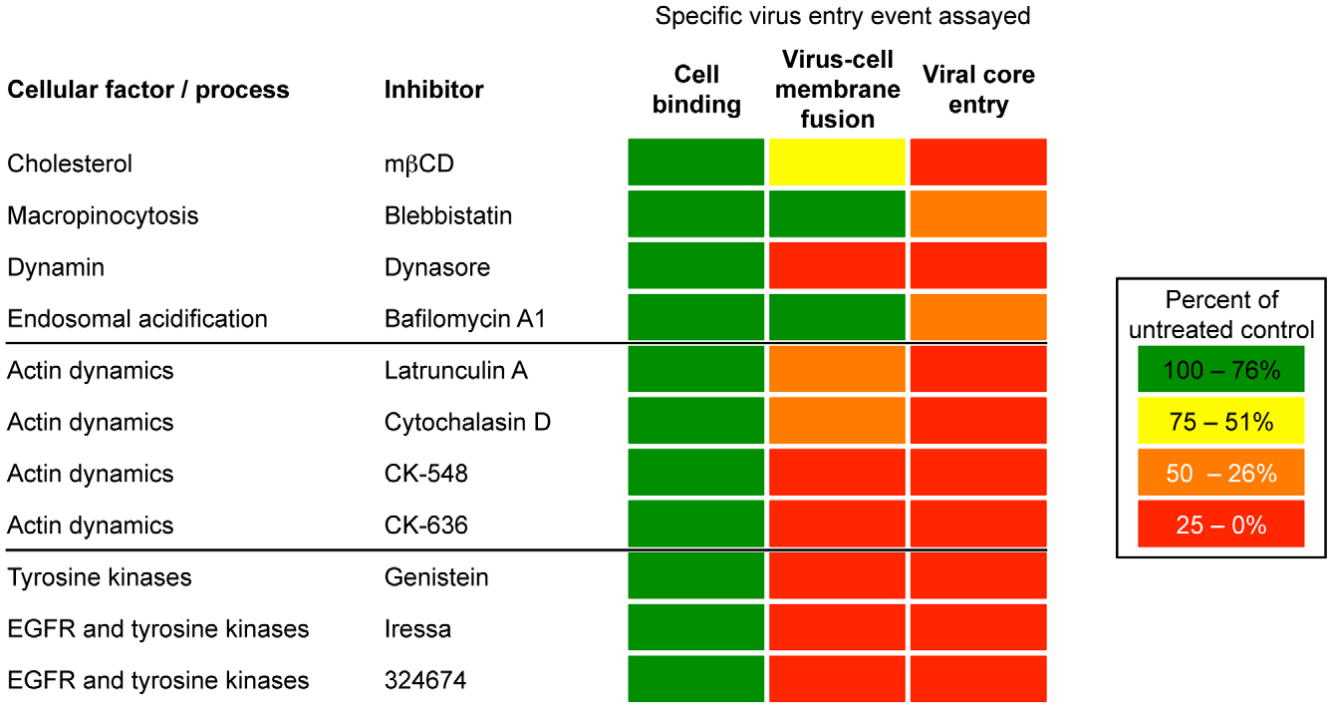
Summary of effects of inhibitors on VACV entry. Cell binding, virus-cell membrane fusion, and viral core entry were assessed as described in the text and as depicted in Figures 1 and 3. The inhibitors are grouped according to their best-characterized effects but may also perturb cells in other ways.

In the first part of the Results, we described the effects of inhibitors of cell processes on virion attachment, membrane fusion and core entry. Most of the inhibitors had previously been shown to reduce entry as determined by reporter gene expression or detection of cytoplasmic cores [11]–[13], [16], [42]. We found that none of these inhibitors prevented binding of virions to cells, many reduced membrane fusion, while others only acted at the core entry step (Figure 9). The membrane fusion inhibitors were either directly involved with actin polymerization or remodeling (CK-636, CK-548, latrunculin A, cytochalasin D) or blocked tyrosine kinases that can modulate actin cytoskeletal changes (genestein, Iressa, 324674). The action of dynasore, a specific inhibitor of dynamin GTPase, could be due to its known effect on actin since there is evidence against a role for caveolae-mediated endocytosis in VACV entry [16]. Further evidence for dynamin2 in VACV core entry has been obtained with siRNA [16]. Extensive actin remodeling and mobilization has been observed during MV binding to cell surfaces [11], [16], [42] suggesting that actin-enriched membrane protrusions increase the intimacy of membrane contact and promote virus-cell membrane fusion. Actin remodeling has been suggested to facilitate fusion by forcing membranes together and enlarging pores in a variety of systems [62]–[64] including virus entry and viral protein-induced cell-cell fusion [65]–[70]. With human immunodeficiency virus, actin remodeling appears to have a more important role in pore expansion and content mixing than in hemifusion [71], [72]. We found that cytochalasin D and latrunculin A had a greater inhibitory effect on core entry (determined by LUC expression) than membrane fusion as determined by lipid mixing, suggesting that actin dynamics may be required for multiple steps in VACV entry.

In contrast to the role of actin rearrangement, inhibitors that prevented membrane blebbing involved in virus surfing and macropinocytosis or that interfered with the reduction in pH of endosomes, had a much greater effect on core entry than membrane lipid mixing (Figure 9). It will be important to determine whether lipid mixing is occurring at the plasma membrane or in endosomes at neutral pH. Similarly, a 74% reduction of cellular cholesterol with mßCD had little effect on membrane fusion but had a major effect on core entry as measured by LUC expression. A previous study had shown that MVs associate with cholesterol-rich regions of the plasma membrane and that cholesterol depletion reduced VACV entry as measured by visualizing cores in the cytoplasm [41]. In studies with influenza virus and Semliki Forest virus in insect cells, which can be more stringently depleted of cholesterol than mammalian cells, both hemifusion and pore widening were affected [73], [74]. The cell surface receptors for certain viruses reside in cholesterol-rich lipid rafts, but receptors for VACV have not been identified.

The VACV EFC proteins were previously shown to be required for virus core entry and cell-cell fusion but evidence for a role in the fusion of viral and cell membranes had been indirect. Of the ten EFC or EFC-associated mutants tested in the present study, all were blocked in core entry as determined by infectivity or transmission electron microscopy and seven of these were unable to mediate membrane fusion. The three proteins apparently not required for membrane fusion were A28, L1, and L5. It is possible that these proteins have a specific role at a later step in entry such as pore formation. However, in other systems it has been shown that the density of activated fusion proteins has to be higher for the formation and expansion of a fusion pore than for hemifusion [1]. Although these three mutants each display stringent repression of EFC protein expression as shown by Western blotting, undetectable differences could affect the sensitive lipid-mixing assay. Therefore, our main conclusion is that the EFC is required for membrane fusion and that additional studies are required to conclude that A28, L1 and L5 have a specific role at a later step of entry such as pore formation.

The L1 protein is a target of potent neutralizing and protective antibodies [56], [75]. The structure of L1 alone and in association with a conformation-specific MAb has been solved to high resolution [76], [77]. The Fab fragment binds to a discontinuous epitope containing two loops that are held together by a disulfide bond. Here we showed that the MAb prevents VACV entry at a step beyond lipid mixing, consistent with the effect on entry of virions deficient in the L1 protein.

Since our inhibitor studies had shown that actin dynamics are required for membrane fusion and core entry, we considered the possibility that the EFC indirectly promotes entry by inducing cell signaling. Indeed, such a role could contribute to the need for multiple EFC proteins. Since Mercer and Helenius [11] had shown that cell signaling requires few virus particles, we tried to rescue EFC protein-deficient virions in trans by coinfecting with wild type VACV. Although wild type virus enhanced core entry by four-fold as measured by LUC expression, this value was still only 3% of that achieved by the control virus, suggesting that the EFC proteins have a direct role in membrane fusion and entry. Nevertheless, whether EFC protein interactions also cause signaling is an interesting question for future studies.

Why so many different proteins are needed for poxvirus entry remains an enigma. None of the proteins resemble type I or type II viral fusion proteins by sequence so that determination of the 3-dimensional structure of the VACV EFC may be needed to define putative fusion loops, if the mechanism of entry involves such structures. At this time, only the structure of the L1 EFC-associated protein has been solved [76].

## Materials and Methods

### Cells and Viruses

African green monkey kidney BS-C-1 and human HeLa cells were maintained in minimum essential medium with Earle's salts (EMEM) supplemented with 2.5% fetal bovine serum (FBS), 2 mM L-glutamine, 100 U/ml penicillin, and 100 µg/ml streptomycin (Quality Biological). The recombinant VACV WRvFire expressing firefly LUC under a synthetic early/late VACV promoter was described previously [6]. Recombinant VACVs in which expression of individual EFC or EFC-associated proteins are IPTG-inducible have been previously constructed and characterized: A16 [23], A21 [24], A28 [25], G3 (A. Townsley and BM, unpublished), G9 [28], H2 [29], J5 [31], L5 [34], O3 [33], L1 [32], and F9 [26]. The recombinant VACV in which the I5L gene was deleted has been described [54]. The recombinant VACV Gauss-A4 (parental strain WRvFire), which expresses the Gaussia LUC enzyme fused to the A4 core protein was generated as follows. Overlap polymerase chain reaction (PCR) was utilized to generate a construct in which the Gaussia LUC gene (New England Biolabs) was appended to the N-terminal codon of the VACV A4L gene and the EGFP coding region (and accompanying synthetic early/late VACV promoter sequence) was placed downstream of the Gaussia-A4L region. To achieve homologous recombination, flanking genomic sequences of A4L (approximately 500 bp in length) were appended to the termini of the PCR product. HeLa cells were infected with 0.05 PFU of WRvFire per cell and at 2 h post infection were transfected with 400 ng of purified PCR product using Lipofectamine 2000 (Invitrogen) according to the manufacturer's protocol. At 24 h post infection, the infected cells were lysed by five freeze/thaw cycles and clonally purified five times by picking GFP positive plaques on BS-C-1 cells. The recombinant VACV in which A28L is IPTG-inducible and expresses firefly LUC under a synthetic early/late VACV promoter has been described [25].

### Purification and Quantitation of Virus Particles

BS-C-1 cells were infected with VACV in the presence or absence of the inducer IPTG (Calbiochem) and at 48 to 72 h post infection MVs were isolated as described [78], [79]. Briefly, infected cells were subjected to Dounce homogenization and MVs were purified by sedimentation through two 36% (wt/vol) sucrose cushions followed by one sedimentation on a 25 to 40% (wt/vol) continuous sucrose gradient; the visible virus band was collected, and virus was pelleted and stored at −80°C. Upon thawing, virus was sonicated on ice for 1 min. The infectious viral titer (PFU per ml) for each purified MV stock of recombinant VACV was determined by plaque assay on BS-C-1 cells as described [80]. Additionally, the number of total virus particles obtained for each purified MV stock of recombinant VACV was estimated from the optical density at 260 nm [80].

### R18 Loading of Virus Particles and Fusion Assay

Purified MVs (approximately 9.0×10^9^ particles) were labeled with 3 ml of 1 mg/ml of R18 (Molecular Probes) in phosphate-buffered saline (PBS; Quality Biological) + 0.2% bovine serum albumin (BSA; Sigma-Aldrich) for 20 min at room temperature in the dark. Non-incorporated R18 was removed by pelleting virions (16,000 x *g* for 10 min at 4°C) and washing several times in PBS + 0.2% BSA. R18-labeled virions were re-suspended in PBS + 0.2% BSA, vortexed, and sonicated for 15 sec on ice. Virions sufficient to achieve a multiplicity of 1 to 5 PFU (or the equivalent number of non-infectious particles) per cell were then incubated with approximately 1.5×10^6^ HeLa cells in suspension for 1 h at 4°C in cold fusion medium comprised of EMEM without phenol red and with 10 mM *N*-2-hydroxyethylpiperazine-*N*'-2-ethanesulfonicacid (HEPES) and 10 mM 2-(*N*-morpholino)ethanesulfonic acid (pH 7.4) in the dark. Virus-bound cells were washed twice with cold fusion medium following low-speed centrifugation (750 x *g* for 3 min at 4°C). Virus-bound cells were injected into a cuvette containing fusion medium pre-warmed to 37°C and kept in suspension utilizing a magnetic stir bar. R18 fluorescence (560 nm excitation and 590 nm emission) was monitored by use of a Fluoro-Max3 spectrofluorometer (Horiba Jobin Yvon) outfitted with a Peltier sample cooler (Horiba Jobin Yvon) and a temperature control unit (Wavelength Electronics model LFI-3751) to maintain the desired temperature within the chamber housing the sample cuvette. For graphical presentation, the raw fluorescence data were plotted versus time. For quantitative comparisons, we determined the percent fluorescence by dividing the value obtained at 40 min by the value obtained following addition of Triton X-100 (1% [wt/vol] final concentration).

### LUC Core Entry Assay

HeLa cells seeded in 24-well plates (2.0×10^5^ cells per well) were chilled to 4°C before virus adsorption. WRvFire MVs were adsorbed in cold EMEM + 2.5% FBS for 1 h at 4°C. Cells were washed with cold PBS to remove unbound virions and incubated with pre-warmed EMEM + 2.5% FBS for 2 h (unless indicated otherwise) at 37°C. Cells were washed with PBS and then incubated with Cell Culture Lysis Reagent (Promega) for 30 min at room temperature with gentle agitation. LUC activity in cellular extracts was measured according to the manufacturer's protocol (Promega) and quantified on a Berthold Sirius luminometer (Berthold Detection Systems).

### Cholesterol Depletion of Target Cells

HeLa cells seeded in 24-well plates (2.0×10^5^ cells per well) were left untreated or treated with 10 mM mßCD (Sigma-Aldrich) for 30 min in EMEM at 37°C. Cells were then washed with cold PBS and cold EMEM was added to cells prior to virus adsorption at 4°C for R18 hemifusion or LUC entry assays as described above. Cholesterol levels in HeLa cells were determined using the Amplex Red Cholesterol Assay Kit (Molecular Probes) and was performed according to the manufacturer's protocol. The viability of mßCD-treated cells was assayed using the CellTiter 96 Aqueous One Solution Cell Proliferation Assay (Promega) and was performed according to the manufacturer's protocol.

### Inhibitor Treatments

HeLa cells were left untreated or pre-treated with the indicated concentrations of inhibitors: Sigma-Aldrich: blebbistatin (75 µM), dynasore (100 µM), bafilomycin A1 (50 nM), latrunculin A (10 µM), cytochalasin D (10 µM), CK-548 (100 µM), CK-636 (100 µM), genistein (100 µM); LC Laboratories: Iressa (40 µM); EMD4Biosciences: 324674 (40 µM) for 30 min at 37°C. Cells were then chilled to 4°C prior to virus adsorption for virus-cell binding, R18 hemi-fusion, or LUC assays as described. The indicated drug concentrations were maintained throughout the assay.

### Virus-Cell Binding Assay

Equivalent amounts of VACV Gauss-A4 virions (5 PFU per cell) were incubated with untreated or inhibitor-treated HeLa cells in 24-well plates at neutral pH for 1 h at 4°C. Cells were washed twice with cold PBS to remove unbound virus. Cells were then incubated with LUC assay lysis buffer (Promega) for 30 min at room temperature with gentle agitation. Gaussia LUC activity in cellular extracts was measured according to the manufacturer's protocol (Promega) and quantified on a Berthold Sirius luminometer (Berthold Detection Systems).

### Stimulation of Virus Entry by Low pH Treatment

Low pH stimulation of virus entry was performed as described previously [6]. Following a wash to remove unbound virions, cells were incubated for 3 min in 37°C PBS with Ca^2+^ and Mg^2+^ at pH 7.4 or PBS with Ca^2+^ and Mg^2+^ supplemented with 1 mM 2-morpholinoethane-sulfonic acid adjusted to pH 5.0 with HCl. After removal of buffers, the pH was neutralized by one wash with EMEM + 2.5% FBS. Cells were incubated in pre-warmed EMEM + 2.5% FBS for 2 h at 37°C and then prepared for the LUC entry assay as described above.

### Transmission Electron Microscopy

BS-C-1 cells in six-well tissue culture plates (1.0×10^5^ cells per well) were pre-chilled at 4°C for 30 min prior to virus spinoculation. Purified MVs (350 PFU per cell or equivalent number of particles) in cold EMEM + 2.5% FBS were sedimented onto the BS-C-1 cells at 4°C for 1 h at 650 x *g* in a Legend RT centrifuge (Sorvall). Cells were washed with cold PBS to remove unbound virions and incubated with pre-warmed EMEM + 2.5% FBS for varying amounts of time at 37°C. At the indicated time, the samples were fixed on ice with 4% paraformaldehyde (Electron Microscopy Sciences) in 0.1 M phosphate buffer for 10 min and processed for transmission electron microscopy as described previously [6]. For quantitation of virus entry events, ninety randomly selected cell sections were visualized and particles therein counted.

### MAb Neutralization

Equivalent numbers of R18-loaded MV particles (recombinant strain WRvFire) were incubated with 100 µg/ml of anti-L1 mouse MAb 7D11 [56] or control anti-HA mouse monoclonal (clone 16B12, Covance) for 30 min at room temperature. Virion and antibody mixtures were then divided and used for R18-based fusion, LUC core entry, or plaque formation assays as described above.

### DiD Loading of Virus Particles and Fusion Assay

Purified MVs (approximately 9.0×10^9^ particles) were labeled with 3 µl of DiD (Molecular Probes) in phosphate-buffered saline (PBS; Quality Biological) + 0.2% bovine serum albumin (BSA; Sigma-Aldrich) for 20 min at room temperature in the dark. Non-incorporated DiD was removed by pelleting virions (16,000 x *g* for 10 min at 4°C) and washing several times in PBS + 0.2% BSA. DiD-labeled virions were re-suspended in PBS + 0.2% BSA, vortexed, and sonicated for 15 sec on ice. Virions sufficient to achieve a multiplicity of 1 to 5 PFU per cell were then incubated with approximately 8.0×10^4^ HeLa cells in a 48-well plate for 90 min at 37°C in minimum essential medium with Earle's salts (EMEM) supplemented with 2.5% FBS, 2 mM L-glutamine, 100 U/ml penicillin, and 100 µg/ml streptomycin. Cells were washed with PBS, trypsinized, spun and fixed in 4% paraformaldehyde/PBS for 2 h at 4°C. DiD-positive cells were quantified using a FACSCalibur (BD Biosciences). DiD loading had minimal effect on virus infectivity as measured by plaque assay.

## Supporting Information

Figure S1 **Effects of mßCD treatment of cells on cholesterol levels and cell viability.** HeLa cell monolayers were left untreated or treated with 0 to 10 mM mßCD for 30 min at 37°C. (A) Cholesterol levels of mßCD-treated cells were determined as described in Materials and Methods. (B) Viability of mßCD-treated cells was assayed using the CellTiter 96 Aqueous One Solution Cell Proliferation Assay (Promega) according to the manufacturer's protocol and plotted as arbitrary units. The assay background value (b.g.) is indicated.
(PDF)

Figure S2 **Attempt to bypass effects of inhibitors by brief low pH treatment.** (A – C) Equivalent numbers of WRvFire MVs were adsorbed to control and inhibitor-treated (latrunculin A (lat.A), cytochalasin D (cyto.D), dynasore, or genistein) cells at 4°C for 60 min. Cells were washed, incubated for 3 min in 37°C PBS Ca^++^/Mg^++^ pH 7.4 or pH 5.0 buffers, and incubated in 37°C media at neutral pH for 2 h at 37°C. Cells were then processed for determination of LUC expression. LUC arbitrary units are shown on the y-axis and concentrations of inhibitors on the x-axis. (D and E) Untreated and inhibitor-treated cells were incubated with equivalent numbers of R18-loaded WRvFire MVs at 4°C for 60 min. Washed cells were then incubated at 37°C for 3 min at neutral pH or pH 5.0 while R18 fluorescence was recorded. After 3 min, cell media was adjusted to pH 7.4 as described in Materials and Methods; R18 fluorescence was monitored for the next 37 min. Virus-bound untreated control cells at neutral pH incubated exclusively at 4°C (4°C cntrl) served as a negative control and as described for Figure 2B. The extent of virus-cell membrane fusion was calculated by dividing the R18 fluorescence observed at 40 min for each sample by that of the untreated cell pH 7.4 control value at that time.
(PDF)

Figure S3 **Effects of anti-L1 MAb on virus-cell membrane fusion, viral core entry and virus infectivity.** Equivalent numbers of DiD-loaded virions (WRvFire) were incubated with or without increasing amounts (four-fold dilutions) of either anti-L1 mouse MAb 7D11 or control anti-HA mouse MAb (clone 16B12, Covance) for 30 min at room temperature. Virions were then assayed for ability to mediate virus-cell membrane fusion by DiD dequenching (A) or LUC core entry (B) at 37°C. Virus infectivity (C) was assayed by adsorbing each virus sample at 37°C to BS-C-1 monolayers for 60 min and enumerating plaque formation 48 h later. Data are represented as percent of the no MAb control for each assay.
(PDF)
