# Supplementary figures and images for: The Membrane Fusion Step of Vaccinia Virus Entry Is Cooperatively Mediated by Multiple Viral Proteins and Host Cell Components
Source: PLoS Pathog. 2011 Dec 15;7(12):e1002446. doi: 10.1371/journal.ppat.1002446 (PMC3240603; doi:10.1371/journal.ppat.1002446)

**A**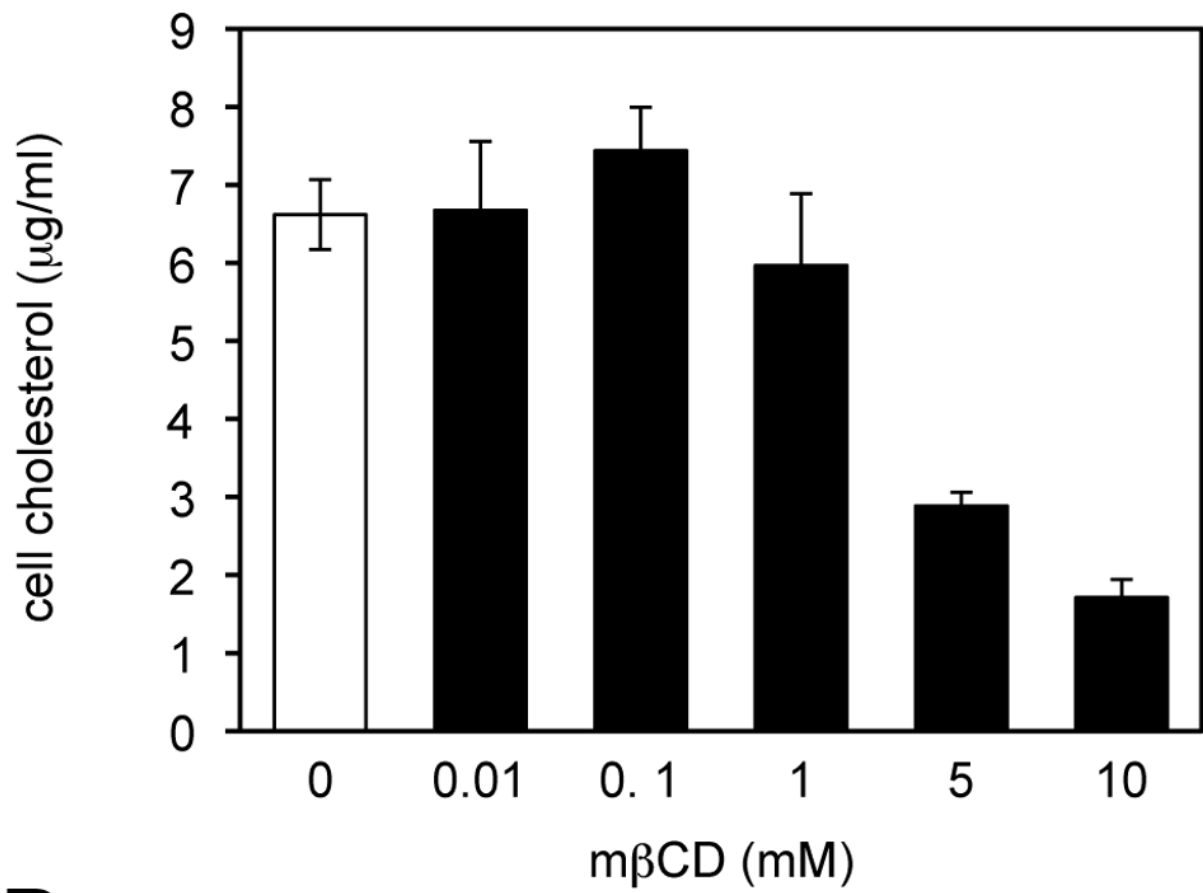**B**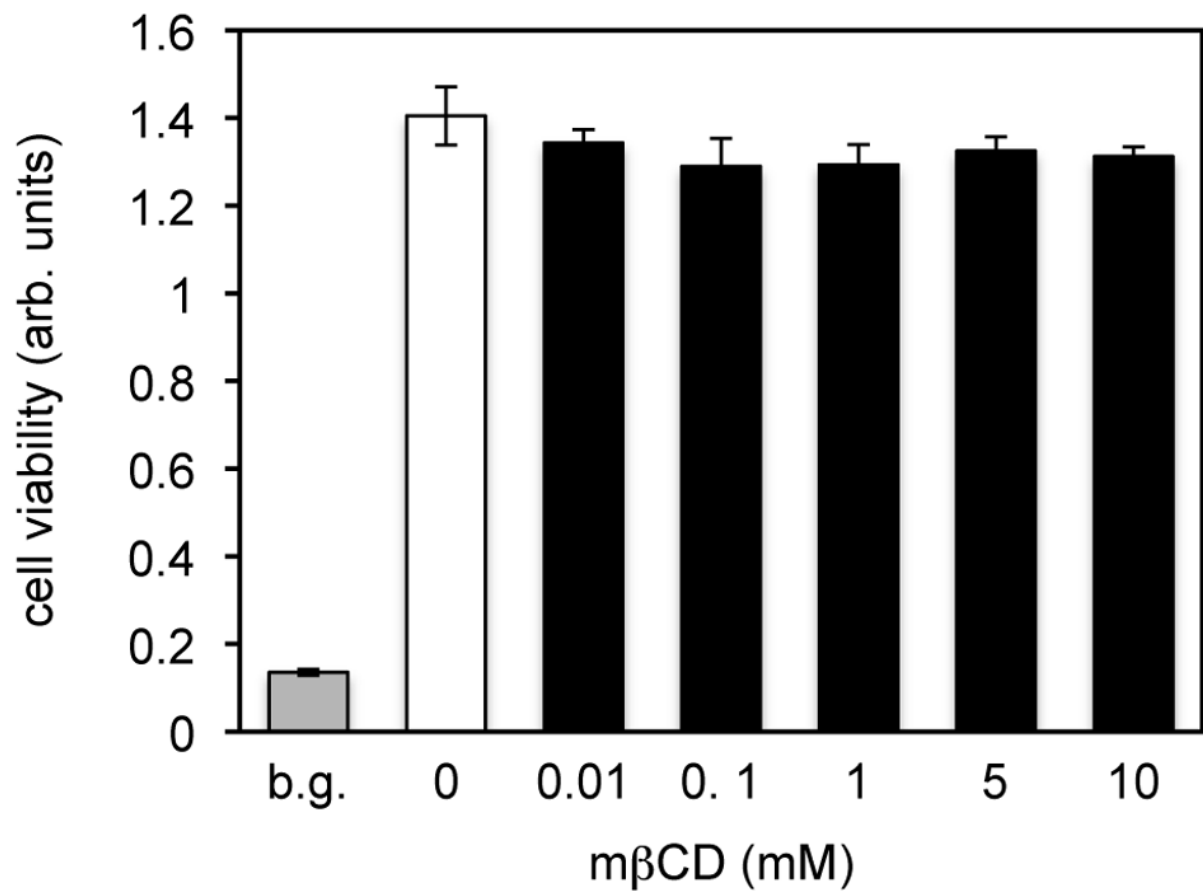

Supplement: Figure S1 — Effects of mßCD treatment of cells on cholesterol levels and cell viability. HeLa cell monolayers were left untreated or treated with 0 to 10 mM mßCD for 30 min at 37°C. (A) Cholesterol levels of mßCD-treated cells were determined as described in Materials and Methods. (B) Viability of mßCD-treated cells was assayed using the CellTiter 96 Aqueous One Solution Cell Proliferation Assay (Promega) according to the manufacturer's protocol and plotted as arbitrary units. The assay background value (b.g.) is indicated. (PDF) [file ppat.1002446.s001.pdf]

**A**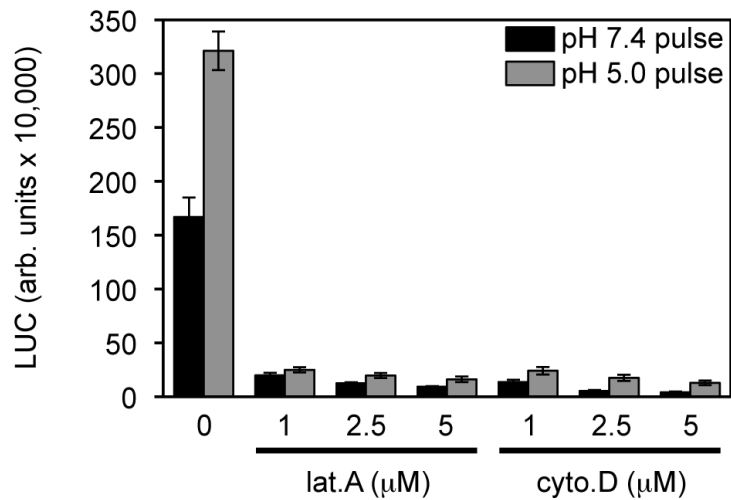**D**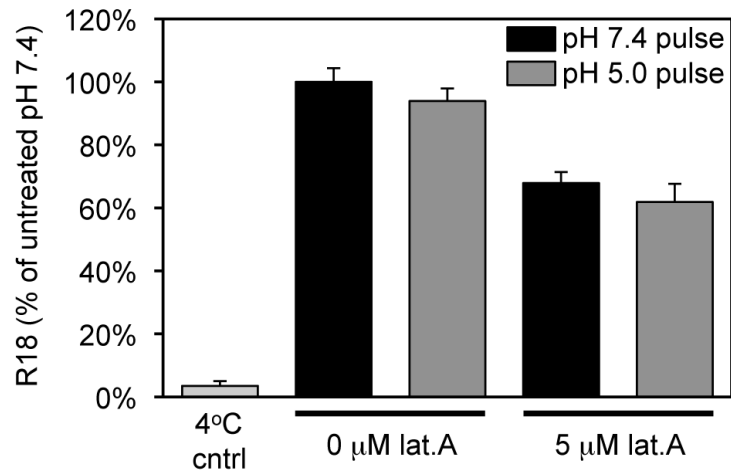**B**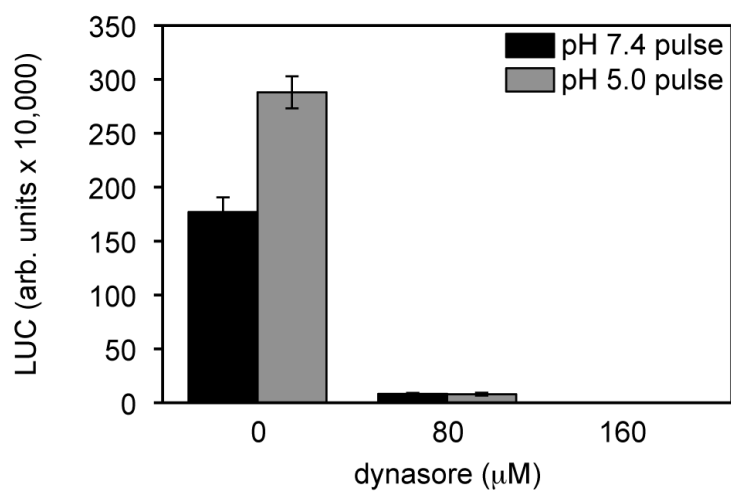**E**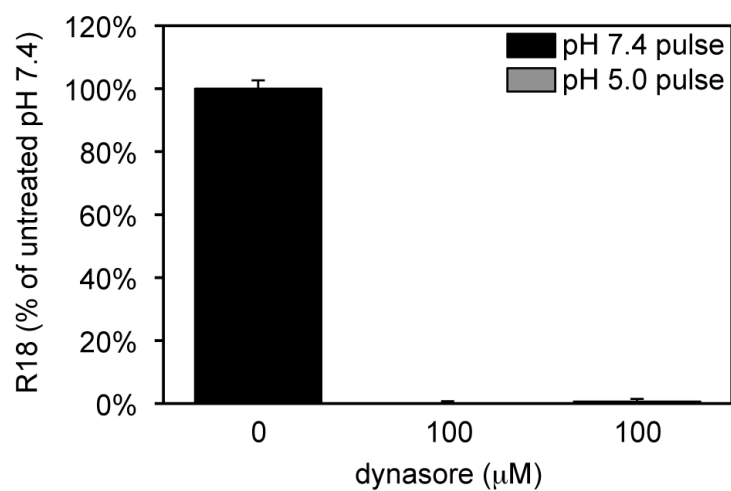**C**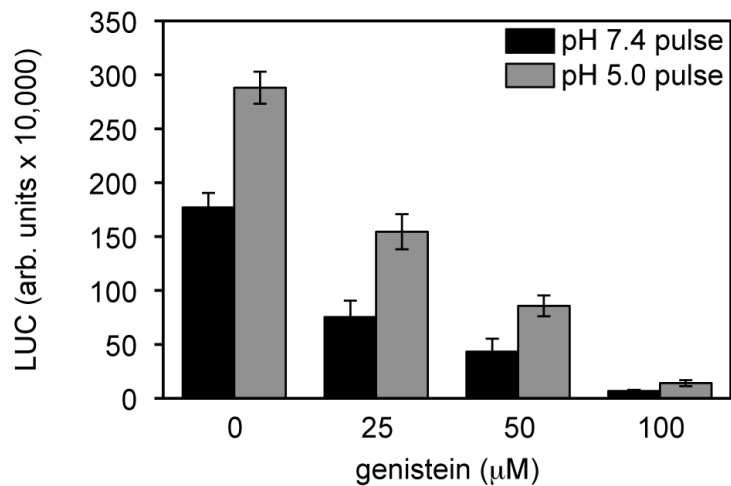

Supplement: Figure S2 — Attempt to bypass effects of inhibitors by brief low pH treatment. (A – C) Equivalent numbers of WRvFire MVs were adsorbed to control and inhibitor-treated (latrunculin A (lat.A), cytochalasin D (cyto.D), dynasore, or genistein) cells at 4°C for 60 min. Cells were washed, incubated for 3 min in 37°C PBS Ca++/Mg++ pH 7.4 or pH 5.0 buffers, and incubated in 37°C media at neutral pH for 2 h at 37°C. Cells were then processed for determination of LUC expression. LUC arbitrary units are shown on the y-axis and concentrations of inhibitors on the x-axis. (D and E) Untreated and inhibitor-treated cells were incubated with equivalent numbers of R18-loaded WRvFire MVs at 4°C for 60 min. Washed cells were then incubated at 37°C for 3 min at neutral pH or pH 5.0 while R18 fluorescence was recorded. After 3 min, cell media was adjusted to pH 7.4 as described in Materials and Methods; R18 fluorescence was monitored for the next 37 min. Virus-bound untreated control cells at neutral pH incubated exclusively at 4°C (4°C cntrl) served as a negative control and as described for Figure 2B. The extent of virus-cell membrane fusion was calculated by dividing the R18 fluorescence observed at 40 min for each sample by that of the untreated cell pH 7.4 control value at that time. (PDF) [file ppat.1002446.s002.pdf]

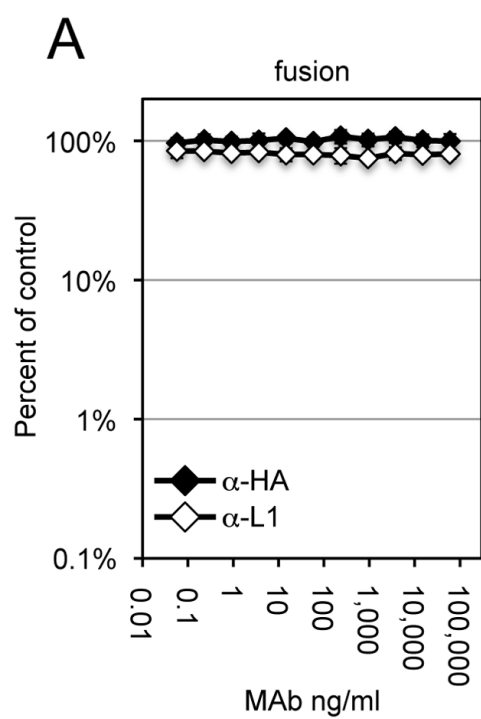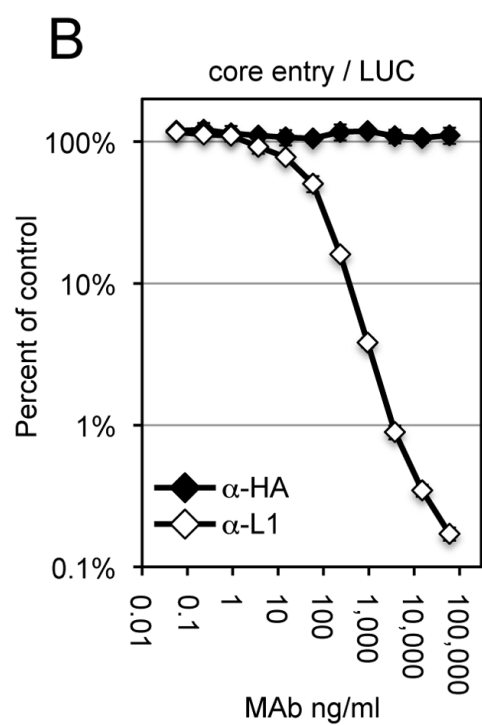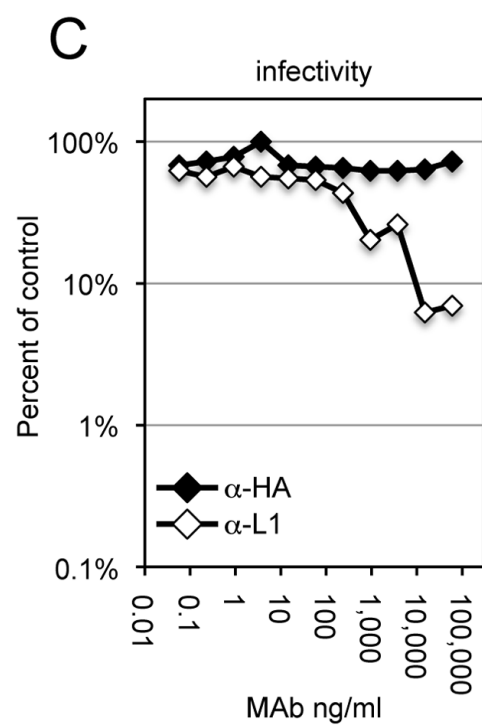

Supplement: Figure S3 — Effects of anti-L1 MAb on virus-cell membrane fusion, viral core entry and virus infectivity. Equivalent numbers of DiD-loaded virions (WRvFire) were incubated with or without increasing amounts (four-fold dilutions) of either anti-L1 mouse MAb 7D11 or control anti-HA mouse MAb (clone 16B12, Covance) for 30 min at room temperature. Virions were then assayed for ability to mediate virus-cell membrane fusion by DiD dequenching (A) or LUC core entry (B) at 37°C. Virus infectivity (C) was assayed by adsorbing each virus sample at 37°C to BS-C-1 monolayers for 60 min and enumerating plaque formation 48 h later. Data are represented as percent of the no MAb control for each assay. (PDF) [file ppat.1002446.s003.pdf]
